# Exploring the potential roles of apelin, visfatin, and irisin in energy regulation in farm animals: an overview

**DOI:** 10.3389/fvets.2024.1435788

**Published:** 2024-07-19

**Authors:** Borhan Shokrollahi, Sun-Sik Jang, Hyun Jeong Lee, Hafiz Ishfaq Ahmad, Abdul Rahman Sesay, Ali Ghazikhani Shad, Salim Morammazi, Sameh A. Abdelnour

**Affiliations:** ^1^Department of Animal Science, Sanandaj Branch, Islamic Azad University, Sanandaj, Iran; ^2^Hanwoo Research Institute, National Institute of Animal Science, RDA, Pyeongchang, Republic of Korea; ^3^Animal Nutrition and Physiology Division, National Institute of Animal Science, Rural Development Administration, Wanju-gun, Republic of Korea; ^4^Department of Animal Breeding and Genetics, University of Veterinary and Animal Sciences, Pattoki, Pakistan; ^5^Department of Animal Science, Njala University, Bo, Sierra Leone; ^6^Department of Animal Science, Saveh Branch, Islamic Azad University, Saveh, Iran; ^7^Department of Animal Science, Faculty of Agricultural and Natural Resources, Persian Gulf University, Bushehr, Iran; ^8^Department of Animal Production, Faculty of Agriculture, Zagazig University, Zagazig, Egypt

**Keywords:** apelin, visfatin, irisin, energy regulation, farm animals

## Abstract

Adipose tissue, both intricate and fundamental to physiological functions, comprises cell types, including adipocytes, pivotal in secreting bioactive peptides known as ‘adipokines.’ Apelin (APLN), Visfatin (VSFTN), and Irisin (IRSN) are novel adipokines involved in regulating energy, carbohydrate, protein, and lipid metabolism. APLN acts as an endogenous ligand for G-protein-coupled receptors, VSFTN is essential in nicotinamide adenine dinucleotide (NAD) biosynthesis, and IRSN is released from skeletal muscle and adipose tissues. Their influence spans various physiological domains, including insulin resistance and sensitivity, cardiovascular functions, angiogenesis, and reproductive systems. This review focuses on the potential roles of APLN, VSFTN, and IRSN in energy regulation mechanisms related to farm animal production. Despite accumulating evidence of their significance, comprehensive understanding is still emerging, with most studies based on model organisms. Thus, there’s a pressing need for targeted research on farm animals. Addressing these knowledge gaps could pave the way for improved health strategies, reproductive efficiency, and productivity in farm animals. Future research should focus on understanding the multifaceted interactions of these adipokines and their implications for promoting sustainable and effective animal production.

## 1 Introduction

Adipose tissue (AT) is not merely a lipid-storage compartment but a complex endocrine organ with significant metabolic functionality (1). It mediates numerous physiological processes, including neuroendocrine modulation, energetic homeostasis, immunological activities, macrophage reconfiguration, and reproductive functions (2). Historically, AT has been classified into three principal categories: white AT (WAT), brown AT (BAT), and beige AT (BeAT) (3). WAT, which is widely distributed throughout the human body, includes mature adipocytes, macrophages, fibroblasts, endothelial cells, and preadipocytes, all playing integral roles in various energetic homeostasis pathways (1, 4). Conversely, BAT is specialized in energy expenditure, contrasting with WAT’s energy storage function. BAT’s distinguishing features include a high content of lipid droplets and an abundance of mitochondria, which facilitate its role in thermogenesis (5).

Notably, WAT secretes a wide array of bioactive peptides, including both anti-inflammatory and pro-inflammatory factors. These peptides, known as ‘adipokines’ or ‘cytokines,’ have diverse roles, such as modulating energetic homeostasis, regulating reproductive processes, adjusting metabolic balance, and being linked to various cardiovascular pathologies associated with adiposity (6). Key adipokines include leptin (LEP), Retinol Binding Protein 4 (RBP4), and adiponectin (ADIPOQ) (7).

While adiponectin and leptin are renowned for their involvement in energy regulation (8), emerging adipokines, notably APLN, VSFTN, and IRSN, reveal their significance in energy metabolism. APLN, an endogenous ligand of the APJ, a member of the orphan G-protein-coupled receptor (GPCR) family, closely resembles the angiotensin-II receptor (9). Fluctuations in the plasma levels of APLN are observed with weight variations, associating it directly with obesity (10). APLN also shows potential in modulating insulin sensitivity and cardiovascular functions (11).

VSFTN, also known as pre-B cell colony-enhancing factor 1 (PBEF1) or Nicotinamide phosphoribosyltransferase (NAMPT), is involved in B-cell maturation and neutrophil apoptosis (12). Predominantly found in visceral AT (13), several studies have explored its correlation with obesity and type 2 diabetes (14).

IRSN, a notable myokine and adipocytokine, functions across various tissues (15). It can upregulate the expression of uncoupling protein 1 (UCP1) in mitochondria, with its levels influenced by diet, exercise, obesity, and pharmacological agents (16, 17).

In farm animals, adipokines such as APLN, VSFTN, and IRSN are crucial for modulating nutrient intake, energy flux, and maintaining metabolic homeostasis (18). These mechanisms are especially important in livestock facing challenges related to negative energy balance (NEB) (19). The literature highlights the impact of these adipokines on various physiological processes, including metabolic dysregulations, angiogenesis, lipid homeostasis, and adiposity. Additionally, they have diverse effects on the mammalian reproductive system (20).

Despite their significance across multiple species, there is a notable gap in understanding their specific roles in the energy metabolism of farm animals. This review aims to investigate the structures, functions, and potential applications of APLN, VSFTN, and IRSN across different species, with a particular focus on farm animals. By comparing their known roles in other species, we emphasize the urgent need for targeted studies on these adipokines in livestock to enhance energy metabolism and production.

## 2 APLN isoforms, gene localization, and molecular interactions

APLN, recognized as an endogenous ligand, uniquely binds to the APLN receptor (APJ) (21). It originates from APLN-77 or pre-pro-APLN. A spectrum of APLN isoforms emerge from it, including APLN-36, APLN-19, APLN-17, APLN-13, and APLN-12 (22–24). These isoforms are derived from the C-terminal of the preproprotein (25, 26). APLN-36 is considered the mature peptide due to its alignment with the 36\u00B0C-terminal amino acids (AAs) of the pre-pro-APLN protein (25). The conserved C-terminal 17 AA sequence, labeled as APLN-17 or K17F, includes APLN-13 (9, 25). Alterations in APLN-13’s N-terminal glutamine result in pyroglutamyl formations, highlighting significant APLN molecular structures (27).

### 2.1 Gene localization

The APLN gene is located on chromosome Xq25-26.1 in humans, marked by a single intron around 6 kb long (9, 28). In rats and mice, the gene is positioned at Xq35 and XA3.2, respectively (9). Identified primary promoter regions include-207/−1 for rats and-100/+74 base pairs for humans (29). A significant similarity of 76–95% is observed among bovine, human, rat, and mouse pre-pro-APLN precursors, with each species believed to possess a native dimeric APLN protein form (30).

### 2.2 APJ receptor

APJ, also designated as APLNR, belongs to the seven-transmembrane G-protein coupled receptors (GPCRs) family, specifically within the Rhodopsin-like GPCRs class (31). Its sequence comprises 380 AAs, and its nomenclature is driven by the interaction with its ligand, APLN (32).

### 2.3 Molecular conformation

Although a definitive APLN structure is absent in the Protein Data Bank (PDB), available studies report that APLN-13 and APLN-36 adopt a “random conformation” at ambient temperatures (33). Circular dichroism spectroscopy (CD) studies indicate a random coil conformation for various APLN isoforms at 5°C and 35°C. APLN-17 is characterized by dominant β-turns and polyproline-II (PPII) structures spanning its peptide configuration (24, 26).

### 2.4 Expression and function

**Figure 1:**
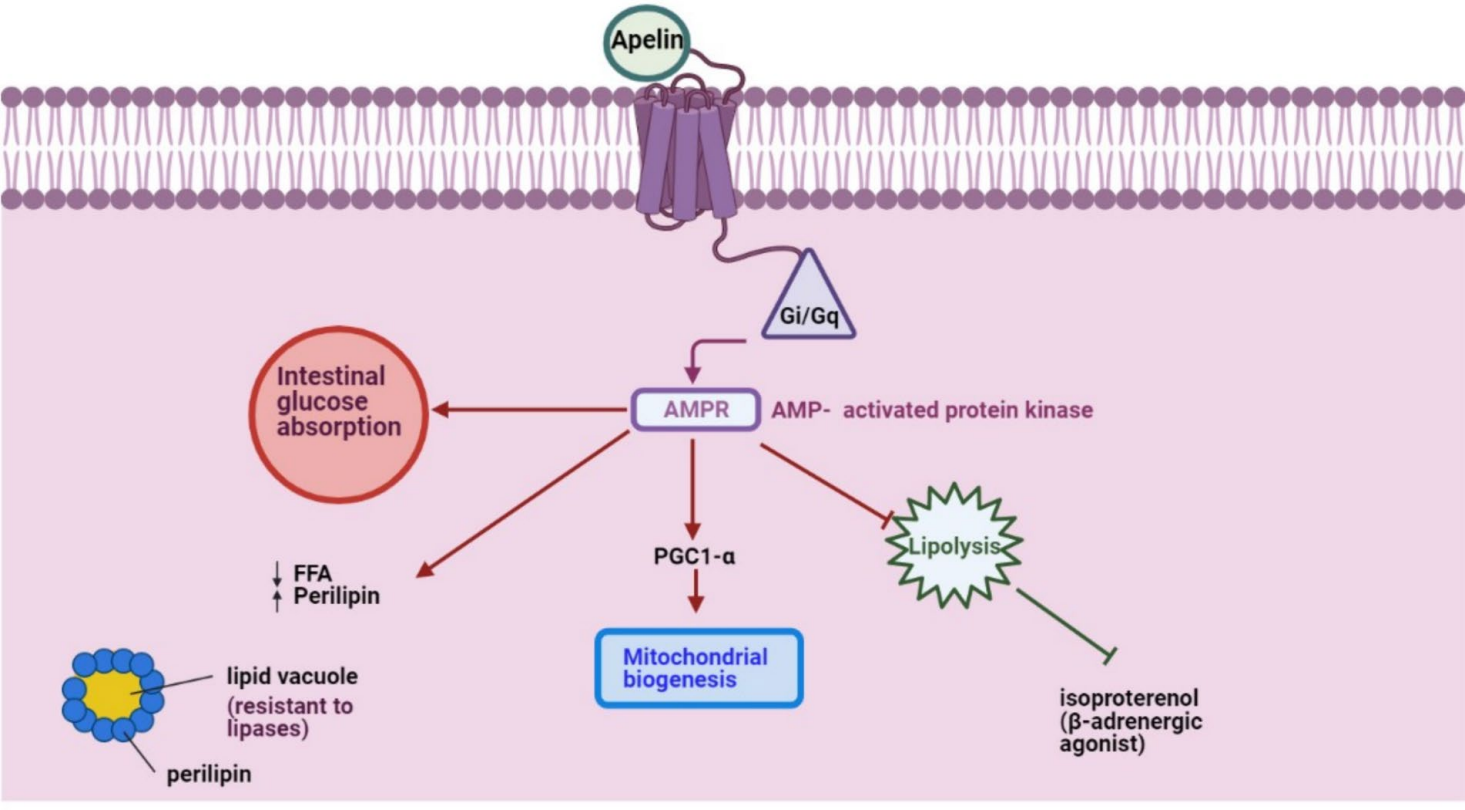
APLN, a GPCR ligand, activates Gi/Gq pathways to modulate intracellular AMPR concentration and inhibit isoproterenol-induced lipolysis, initiating other pathways.

APLN mRNA is detected in stromal-vascular cells and adipocytes (34). However, its expression remains stable across intraabdominal and subcutaneous fat tissues (10). A key functional aspect of APLN is its activation of the Gi/Gq pathways, modulating intracellular AMP concentrations and inhibiting isoproterenol-induced lipolysis among other pathways (Figure 1). Exploring APLN’s structural and functional nuances can illuminate its potential implications in energy regulation within farm animals.

## 3 APLN’s distribution and its integral role in glucose homeostasis and insulin sensitivity

### 3.1 Distribution

APLN is ubiquitous in both humans’ and rats’ central nervous system (CNS) and peripheral tissues (35). It is detected in various organs, such as the testicles, intestines, and fetus, with variable expression levels across the placenta, heart, lungs, kidneys, and vascular endothelial cells (36–38). Distinctly, APLN expression is pronounced across adipose, connective, and vascular smooth muscle cells (36). Additionally, its presence spans various fish tissues, encompassing the brain, pituitary, spleen, kidney, and liver (39–41). In sheep, APLN is also expressed in peripheral organs such as the mammary gland, abomasum, and duodenum, in addition to the uterus (42–44).

### 3.2 Role in glucose homeostasis

Central APLN’s relevance is undeniable. Several CNS nuclei exhibit APLN mRNA, indicating its role in glucose metabolism (45). Specifically, APLN-positive nerve fibers in the hypothalamus point to APLN-producing neurons that modulate glucose homeostasis, influenced by circulating peptides and neurotransmitters (46). Numerous studies have solidified APLN’s critical role in insulin sensitivity and glucose metabolism. For instance, during conditions that inhibit hepatic glucose production, APLN fosters decreased blood glucose levels by enhancing glucose uptake in skeletal muscles and ATs, ultimately boosting insulin sensitivity (47, 48). Mechanistically, APLN promotes glucose transport in muscles and modulates pathways involving AMP-activated protein kinase (AMPK) and endothelial nitric oxide synthase (eNOS) (11, 48). AMPK activation in muscle cells is pivotal for APLN-induced glucose uptake (49). APLN also enhances glucose movement from the intestinal lumen to the bloodstream, influencing glucose levels in the portal vein and insulin production and sensitivity (36, 50).

### 3.3 Findings in dairy cows

Notably, studies on dairy cow reveal contrasting findings on APLN levels post-calving. One study observed decreased APLN levels after calving, with linked fluctuations in insulin and glucose concentrations (51). In contrast, another study reported consistent APLN levels during the transition from pregnancy to lactation (52).

APLN’s widespread distribution and its integral role in glucose metabolism and insulin sensitivity emphasize its potential importance in farm animal health, especially dairy cows. Monitoring APLN fluctuations might be key to optimizing these animals’ metabolic health and energy balance, particularly during pivotal stages such as pregnancy-to-lactation transition.

**Figure 2:**
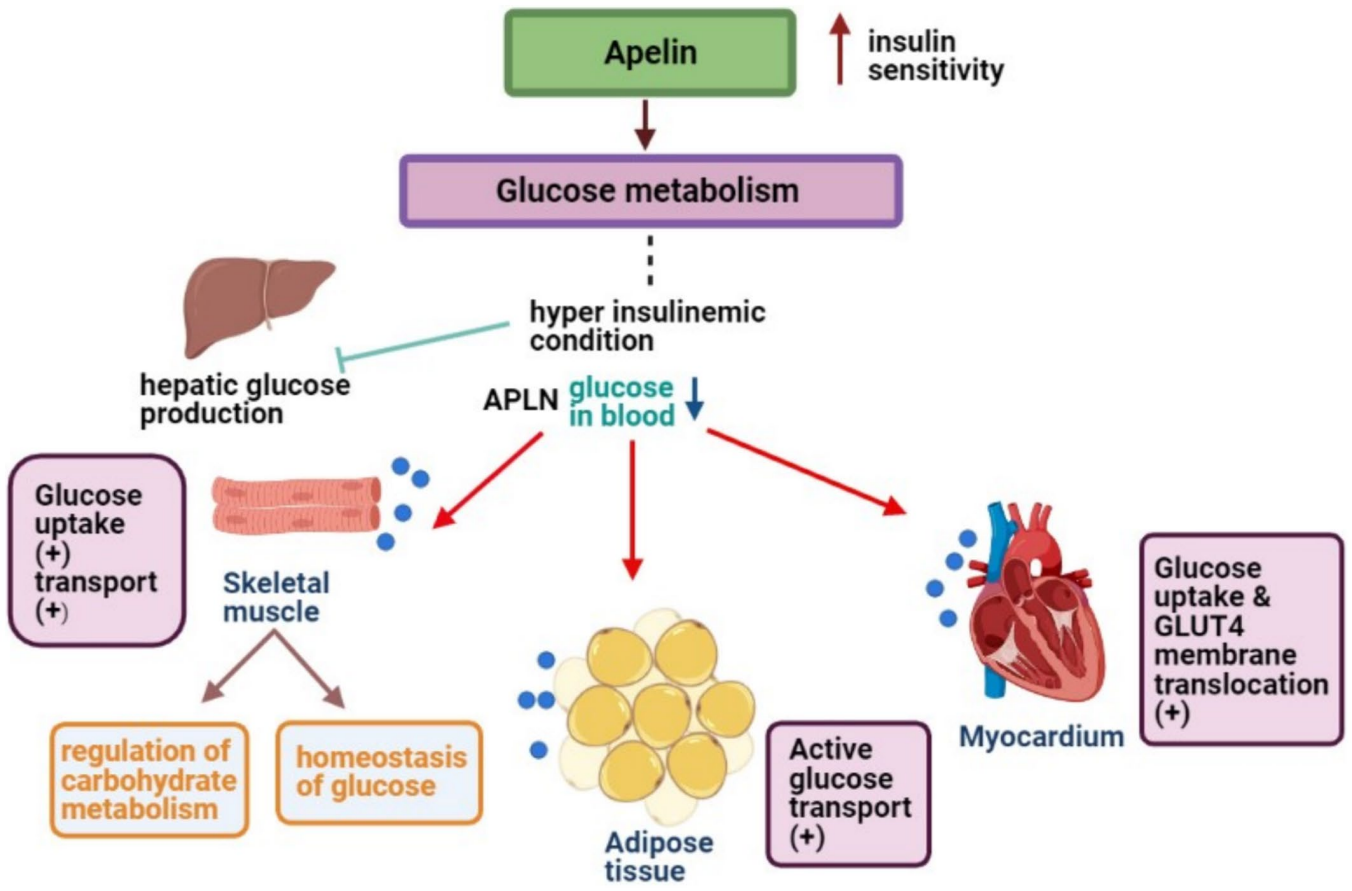
APLN enhances glucose uptake in skeletal muscles, ATs, and myocytes to maintain low blood glucose levels.

For a visual representation of the aforementioned mechanisms, Figure 2 offers a detailed schematic overview.

## 4 APLN’s multifaceted impact on lipid regulation and systemic metabolic functions

### 4.1 Energy homeostasis

Energy homeostasis and nutrient metabolism are harmonized by a synergy of paracrine, endocrine, and autocrine regulators. Vital organs like the liver, pancreatic β-cells, AT, and skeletal muscle orchestrate this balance (53). Imbalances may usher in conditions like obesity, often linked with insulin resistance. Increased plasma APLN concentrations have been associated with obesity and type 2 diabetes (48).

### 4.2 Lipid regulation

APLN counteracts isoproterenol-induced lipolysis in isolated and differentiated 3 T3-L1 adipocytes (54). This modulation leverages the GQ, Gi, and AMPK signaling pathways, crucial for orchestrating cellular metabolic responses (55). By inhibiting lipid catabolism in adipocytes, APLN limits the release of free fatty acids. This is achieved through AMPK activation and the upsurge in perilipin surrounding lipid vacuoles, shielding stored lipids from lipase activity (54). APLN also fosters insulin secretion and enhances mitochondrial biogenesis in skeletal muscles and cardiomyocytes, increasing mitochondrial density and efficiency (56, 57).

### 4.3 Role in angiogenesis and fluid homeostasis

APLN promotes endothelial cell proliferation, migration, and neovascularization (45). In the hypothalamus, APLN regulates the secretion of antidiuretic hormone (ADH), essential for fluid homeostasis (58). It also triggers α-MSH release, a neuropeptide known to reduce food consumption, potentially offsetting metabolic disparities in obese subjects despite receptor desensitization (59). APLN and its receptor, APJ, play roles in water metabolism, synergizing with ADH to maintain fluid balance (36).

### 4.4 Implications for farm animals

APLN’s role in lipid and broader metabolic processes highlights its potential significance for livestock metabolic health. By influencing lipid metabolism, enhancing mitochondrial activity, and promoting angiogenesis, APLN could be pivotal in optimizing livestock productivity and overall health.

A summary of APLN’s metabolic impacts, as documented in reported findings from different species, is presented in Table 1.

**Table 1:** Biological effects of APLN in animals: a summary of findings.

Name	Dose	Species	Mode of administration	Major effect	Reference
APL APLN-13	10 μg, 100 μg and 1 mg at 1-h intervals.	Ewe	Intravenous boli injections	Injection of APLN-13 increased the circulation level of several vasoactive hormones, including plasma arginine vasopressin, adrenocorticotrophin, aldosterone, cortisol, atrial and brain natriuretic peptide, cyclic GMP, and cyclic AMP with no effect on renal indices.	(60)
APLN-12, APLN-36 and Pyr^1^-APLN-13	4 μg/kg, 20 μg/kg, and 100 μg/kg	Male Wistar rats	Intravenous bolus injection	APLN-12 (20–100 g/kg) enhanced stomach gastric acid secretion, with a maximum increase of 203% at 100 g/kg (n = 5).	(61)
APLN-13 and APLN-13(F13A)	0.3, 1, 3 and 10 μg/mouse	Male Kunming mice	Intracerebroventricular injection	Injections of APLN-13 (3 and 10 g/mouse) intracerebroventricular lowered the stomach emptying rate by 10.9 and 17.1%, respectively.	(62)
APLN-13	IV injection of 10 nmol and ICV injection of 1 and 3 nmol	Male Wistar rats	Intravenous (IV) and intracerebroventricular (ICV) injection	Injecting 1 or 3 nmol of APLN-13 into the intracerebroventricular space of both fed and fasting rats reduced their food intake.	(63)
APLN-13	0.1 μmol/kg body wt^_^1/day^_^1	Male Wistar rats	Intraperitoneal injection	The nuclear and mitochondrial respiratory chain subunits were elevated in the triceps of APLN-13-injected rats. Likewise, APLN treatment boosted the protein level of mitochondrial import and assembly pathway components.	(64)
APLN-13	40 and 400 pmol/kg	Sprague Dawley male rats	Intravenous injection	Injection of APLN-13 intravenously decreased blood glucose and urine protein levels.	(65)
APLN-13	2 lg, i.c.v.	DIO male rats	Intracerebroventricular injection	Injection of APLN-13 into the intracerebroventricular space decreased food and water intake and respiratory exchange ratio in rats fed a normal diet but had no effect on rats fed a high-fat diet.	(66)
APLN-13 pyroglutamated APLN-13	2 mg/kg/day And 100 nM	C57BLK6/J mice	Intravenous injection	Injecting APLN-13 intravenously into C57BL6/J mice boosted myocardial glucose absorption and GLUT4 membrane translocation. APLN was also adequate for enhancing glucose absorption in H9C2 cells. AMPK inhibition dramatically reduced the glucose absorption mediated by APLN. Finally, APLN enhanced the phosphorylation of IRS-1 Ser-789 in an AMPK-dependent way.	(67)
Pyr-APLN-13	100 mL	Male C57BL/6 J mice	Orally loaded	Pyr-APLN-13 improves glucose utilization in normal and obese/diabetic mice by lowering ENS/contraction activity, which increases hypothalamic NO release. As a result, glucose entry into the muscle is greatly boosted.	(68)
Pyr-APLN-13	0.1 μmol/kg/day	Mice C57BL/6 J,	Intraperitoneal injection	Injection of Pyr-APLN-13 into the intraperitoneal cavity increases brown adipocytes’ differentiation, metabolic activity, and white adipocytes’ browning.	(69)

cGMP, Cyclic guanosine monophosphate; cAMP, Cyclic adenosine monophosphate; GLUT4, Glucose transporter type 4; AMPK, AMP-activated protein kinase; ENS, Enteric nervous system; NO, Nitric oxide.

## 5 Diverse signaling pathways and physiological impacts of APLN in metabolic regulation

### 5.1 Signaling pathways

APLN modulates a host of cellular activities through distinct signaling pathways, notably activating AMPK, which reduces the release of free fatty acids (FFA) from 3 T3-L1 adipocytes by enhancing lipoprotein stability (70). Isoforms of APLN, including APLN-36, APLN-13, APLN-17, and [pyr1]-APLN-13, inhibit forskolin-induced cAMP production via the Gαi/o protein in the APLN/APJ system, blocking specific PKA pathway effects (36, 71).

APLN also stimulates glucose transport in human AT explants through the AMPK pathway, while in 3 T3-L1 adipocytes, it enhances glucose transport via the PI3K/Akt signaling pathway (72, 73). Additionally, APLN boosts insulin-stimulated glucose transport in insulin-resistant 3 T3-L1 cells (73).

### 5.2 Physiological impacts

APLN regulates various physiological functions, including blood pressure, energy metabolism, blood flow, hydration, food intake, and immune function (28). It induces nitric oxide (NO) release, leading to vasorelaxation and increased heart muscle contractility (74). APLN also directs endothelial cell chemotaxis and provides anti-apoptotic protection (75). Its interaction with appetite-regulating hormones, such as orexin, highlights its role in complex metabolic networks (76, 77). In sheep, APLN expression has been identified in various peripheral organs, including the mammary gland, abomasum, and duodenum, in addition to the uterus. This widespread distribution suggests that APLN may play multiple roles in energy regulation and reproductive functions in sheep (42). For instance, the presence of APLN in the mammary gland could be associated with lactation and milk production processes. Similarly, its expression in the abomasum and duodenum indicates a potential role in digestive processes and nutrient absorption (43, 44). Further research is necessary to explore these roles and their implications for sheep health and productivity.

### 5.3 Implications for farm animals

Understanding APLN’s multifaceted roles is crucial for energy regulation in farm animals. APLN’s effects on glucose and lipid metabolism, alongside its broader physiological impacts, suggest potential strategies for optimizing energy balance, health, and productivity in livestock. Further research on APLN’s signaling pathways could lead to innovative animal management and health applications.

## 6 Molecular insights and pervasive impact of VSFTN across species and tissues

### 6.1 Identification and structure

VSFTN, initially identified as the pre-B cell colony-enhancing factor (PBEF), is characterized by its 52 kDa molecular weight and 491 amino acids (AAs), which are highly conserved across species (78). The porcine VSFTN gene is located on chromosome SSC9, sharing structural similarities with the human counterpart (13, 79). It plays a crucial role in the nicotinamide adenine dinucleotide (NAD) biosynthesis pathway and is also known as Nicotinamide Phosphoribosyltransferase (NAMPT) (80, 81).

### 6.2 Forms and localization

NAMPT exists in two forms: intracellular (iNAMPT) and extracellular (eNAMPT) (82). iNAMPT is predominantly found in BAT and less in WAT, while eNAMPT is expressed in various cells, including adipocytes, myocytes, neurons, and immune cells (83, 84). iNAMPT is also present in the liver, kidneys, heart, skeletal muscle, and brain (20). eNAMPT, on the other hand, is expressed across a diverse array of cells ranging from adipocytes and myocytes to neurons and immune cells (83).

### 6.3 Structural organization

VSFTN’s monomer is organized into three domains: A, B, and C, each characterized by specific arrangements of α-helices and β-strands (7). Domain A features an antiparallel seven-stranded β-sheet/core flanked by five helices, domain B has another seven-stranded β-sheet/core, and domain C presents a simpler three-stranded β-sheet in an antiparallel configuration (7).

### 6.4 Functional role

VSFTN is integral to NAD biosynthesis and influences pancreatic beta-cell functions (85). As NAMPT, it catalyzes the transformation of nicotinamide into nicotinamide mononucleotide (NMN), a crucial precursor for NAD synthesis. In pancreatic beta cells, this enhances glucose-stimulated insulin secretion through sirtuin-1 activation, a NAD-dependent deacetylase, regulating glucose-responsive insulin secretion (86).

### 6.5 Pervasive presence

VSFTN is detected in diverse human and animal tissues, such as AT, muscle, heart, and bone marrow (87). It has homologs in organisms ranging from mollusks and bacteria to mammals. In mice, VSFTN is located in the hypothalamus and certain pituitary lobes (88). Additionally, there are gender and tissue-specific variations in VSFTN mRNA expression among broiler chickens, indicating its responsiveness to energy balance-related determinants (89).

### 6.6 Implications for farm animals

Given its central role in energy regulation, particularly in NAD synthesis and insulin secretion, VSFTN is pivotal for understanding energy balance in farm animals. Its widespread presence across multiple species and organs suggests that deeper exploration into VSFTN’s mechanisms could unveil strategies to enhance health, metabolic efficiency, and overall productivity in livestock.

## 7 VSFTN’s multifaceted impact: from metabolic regulation to behavioral influence in farm animals

### 7.1 Metabolic regulation

**Figure 3:**
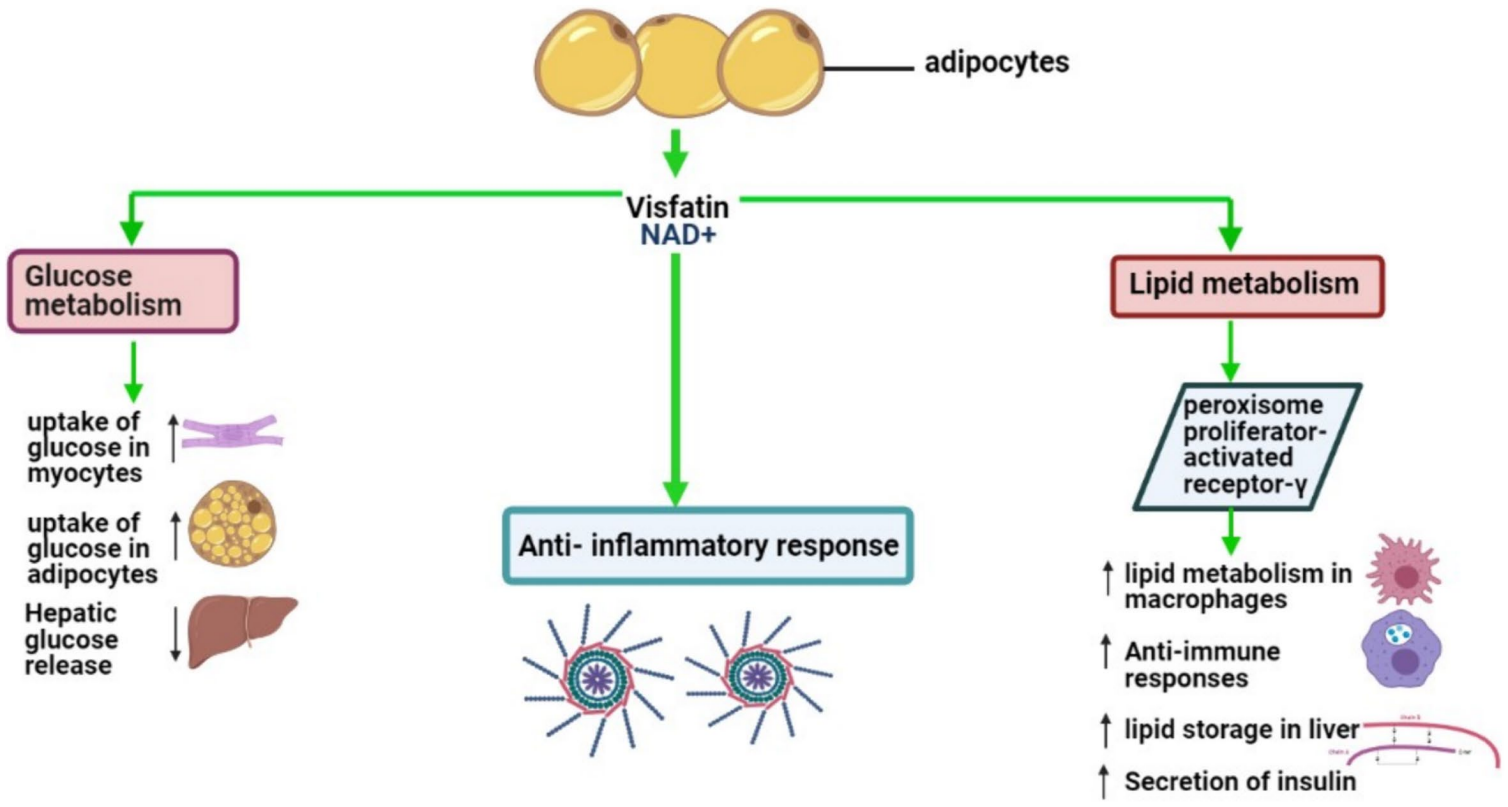
Key biological roles of VSFTN: insights into lipid and glucose metabolism and inflammation.

VSFTN plays an indispensable role in various physiological processes, prominently influencing glucose and lipid metabolism. It significantly contributes to anti-inflammatory responses, glucose and lipid modulation, and behavioral aspects related to food intake (90). In adipocytes, VSFTN-induced NAD biosynthesis acts as a crucial physiological regulator, impacting metabolic activities within AT and exerting systemic influence (91). VSFTN has a metabolic effect analogous to insulin, stimulating glucose uptake in myocytes and adipocytes, augmenting lipogenesis, and suppressing glucose release from hepatocytes (92, 93). A visual representation of these multifarious roles can be found in Figure 3.

### 7.2 Mechanisms

VSFTN’s influence on glucose and lipid metabolism spans endocrine, paracrine, and autocrine pathways, with its autocrine actions notably impacting insulin sensitivity in the liver (92). Research supports these findings; for instance, in a porcine cellular model, VSFTN and insulin elevated lipoprotein lipase and PPARγ mRNA expression in preadipocytes and enhanced fatty acid synthase mRNA expression in differentiated adipocytes (94). Uniquely, VSFTN also increased IL-6 mRNA expression, indicating its involvement in lipid metabolism.

### 7.3 Behavioral influence

VSFTN’s impact extends to behavioral modulation, particularly in regulating food intake and indirectly controlling energy balance (95). For example, an intracerebroventricular injection of VSFTN in chicks increased feed intake and altered brain activity, highlighting its role in modulating feeding behavior (96).

### 7.4 Dietary influence

Dietary inputs can affect VSFTN gene expression. In Hanwoo beef calves, a reduction in concentrate intake increased VSFTN gene expression in the liver, indicating diet’s pivotal role in regulating this gene (97). Furthermore, an inverse correlation between VSFTN expression and body fat levels was observed in pigs, with leaner pigs exhibiting higher VSFTN concentrations, raising questions about its reliability as an indicator of fat storage (98).

The various biological implications of VSFTN across animal studies are systematically summarized in Table 2.

**Table 2:** Summary of findings: biological effects of VSFTN in animals.

Name	Dose	Species	Mode of administration	Major effect	Reference
pcDNA3.1-VSFTN plasmid	300 μg	Male Sprague-drawley rat	Rectus femoris muscle injection	VSFTN/PBEF/NAMPT enhances insulin sensitivity and achieves its hypocholesterolemic effects in part via elevating the tyrosine phosphorylation of IRS-1 protein and the mRNA levels of PPARg and SREBP-2.	(99)
VSFTN	100 pmol	male db/m mice	Intraperitoneal injection	The treatment with VSFTN did not affect body weight, water, feed intake, urine volume, blood glucose, or Hba1c level. VSFTN enhanced HOMAIR, GTT, and ITT while reducing plasma insulin and VSFTN levels but not adiponectin. The plasma levels of cholesterol and triglycerides increased with the VSFTN treatment. The VSFTN significantly reduced albuminuria in diabetic mice. Glomerulosclerosis alteration and mesangial enlargement in the kidneys were significantly diminished. VSFTN also decreased the expression of proinflammatory and profibrotic cytokines, including MCP-1, TGFb1, type IV collagen, and PAI-1.	(100)
VSFTN	40 ng and 400 ng	Roman Brown chicks	Trace stereotactic icv injection	VSFTN dramatically boosted the feed intake of chicks, and glucose, insulin, TG, HDL, and LDL concentrations were significantly altered.	(101)
Recombinant VSFTN	100 μg	Porcine adipocyte cells	Intraperitoneal immunization	VSFTN enhanced lipoprotein lipase expression in preadipocytes, facilitating lipid uptake and increased fatty acid synthase gene expression in differentiated adipocytes, which could enhance lipogenic activity. Moreover, VSFTN can upregulate IL-6 expression in pig adipocytes that have undergone differentiation.	(94)
Recombinant human VSFTN	0.025, and 0.250 nmol	broiler chicks	Intracerebral ventricular (ICV) injection	VSFTN increased chicks’ feed intake and pecking efficiency but did not affect their water intake. C-Fos immunoreactivity was enhanced in the lateral hypothalamus, decreased in the ventromedial hypothalamus, and unaffected in the dorsomedial hypothalamus and infundibular periventricular nucleus, and paraventricular nucleus. A small amount of VSFTN enhanced locomotion.	(96)

GTT, Glucose tolerance test; ITT, Insulin tolerance test; HOMA-IR, Homeostatic Model Assessment of Insulin Resistance; LDL, Low-density lipoprotein; HDL, High-density lipoprotein; TG, Triglycerides.

## 8 Origin, molecular structure, and regulatory mechanisms of IRSN in energy homeostasis

### 8.1 Origin and structure

IRSN, identified as a significant myokine, is released from human muscle tissue following exercise (102). It arises from the precursor protein, fibronectin type III domain 5 (FNDC5), a type-I transmembrane protein initially described in 2002 (103, 104). Structurally, FNDC5 comprises an N-terminal peptide chain (AA sequence 1–28), a Fibronectin-III domain (AA sequence 33–124), a transmembrane domain (AA sequence 150–170), and a cytoplasmic tail (AA sequence 171–209) (105). The extracellular N-terminal tail undergoes proteolytic cleavage, releasing the 112 AA IRSN peptide that circulates across various tissues (102).

### 8.2 Regulatory mechanisms

FNDC5 gene expression is modulated by pivotal proteins like peroxisome proliferator-activated receptor gamma (PPARγ) and coactivator PGC1α (106). PPARγ governs adipocyte differentiation and lipid metabolism, influencing processes such as anti-inflammatory responses, lipid storage in the liver, and glucose-stimulated insulin secretion in pancreatic beta cells (107–109). Conversely, PGC1α is renowned for overseeing mitochondrial biogenesis and regulating oxidative metabolism in diverse cell types (110).

### 8.3 Role in farm animals

Recent studies have further elucidated IRSN’s role in farm animals. One study established that delivery mode affects IRSN concentrations in Holstein calves. Dystocia and cesarean-born calves presented reduced IRSN levels post-colostrum intake, while those born vaginally showed a decline by day 15, highlighting the relationship between birth methods and metabolic adaptations via IRSN concentrations (111). Moreover, IRSN concentrations were notably heightened in cattle with subclinical ketosis compared to their healthy counterparts. This increase was accompanied by a pronounced positive correlation between IRSN and ghrelin levels, indicating IRSN’s potential as a ketosis biomarker (112). Additionally, IRSN has been shown to impact glucose metabolism in cattle by reducing mRNA levels of certain glucose transporters in granulosa cells and increasing lactate release (113).

A positive IRSN-leptin relationship, particularly in cows with subclinical ketosis, aligns with prior studies connecting insulin and IRSN in metabolic disorders (114–117). These findings underscore IRSN’s multifaceted role in farm animals, linking birth methods to IRSN levels and metabolic adjustments. Its elevated concentration in ketotic cattle and associations with ghrelin and leptin further indicate IRSN’s potential as a biomarker for metabolic disorders, specifically subclinical ketosis, and its influence on glucose metabolism.

## 9 Species-specific expressions and uncharted territories of IRSN in farm animals

Recent studies have begun exploring the role and expression of FNDC5 and IRSN in farm animals, revealing differences from humans and mice. For instance, the bovine genome shows higher transcript variability of FNDC5 compared to humans and mice. Although FNDC5 protein distribution remains consistent in bull skeletal muscles, mRNA transcript levels vary significantly in AT and the liver (118). However, detecting IRSN in cattle plasma remains challenging (119).

Further complicating the picture, Daudon et al. (15) posited that FNDC5 and IRSN play roles in lipid mobilization in AT of dairy cattle post-calving. The emerging picture indicates varying and sometimes conflicting views on IRSN’s expression and effects in cattle, warranting deeper investigations. Beyond cattle, the exploration of IRSN’s role in other farm animals has begun. For example, IRSN, FNDC5, and PGC1α were detected in skeletal muscles and WATs of dromedary camels, with correlations observed between their levels and metabolic responses to exercise (120). Further, the IRSN peptide was localized in swine ovaries, suggesting implications for ovarian function (121). Another study on Arabian horses linked serum IRSN levels to exercise regimes (122).

While the above findings shed some light, the overarching theme is clear: Our understanding of IRSN in farm animals, especially its implications on energy regulation, remains limited. Much of the IRSN research focus has historically been on humans and lab models, like mice and rats. This current state presents an extensive opportunity to investigate IRSN’s role in farm animals more comprehensively, aligning with the broader aim of deciphering its potential implications in energy regulation within this context.

## 10 IRSN in metabolic regulation, energy expenditure, and implications for farm animals

### 10.1 Metabolic roles

**Figure 4:**
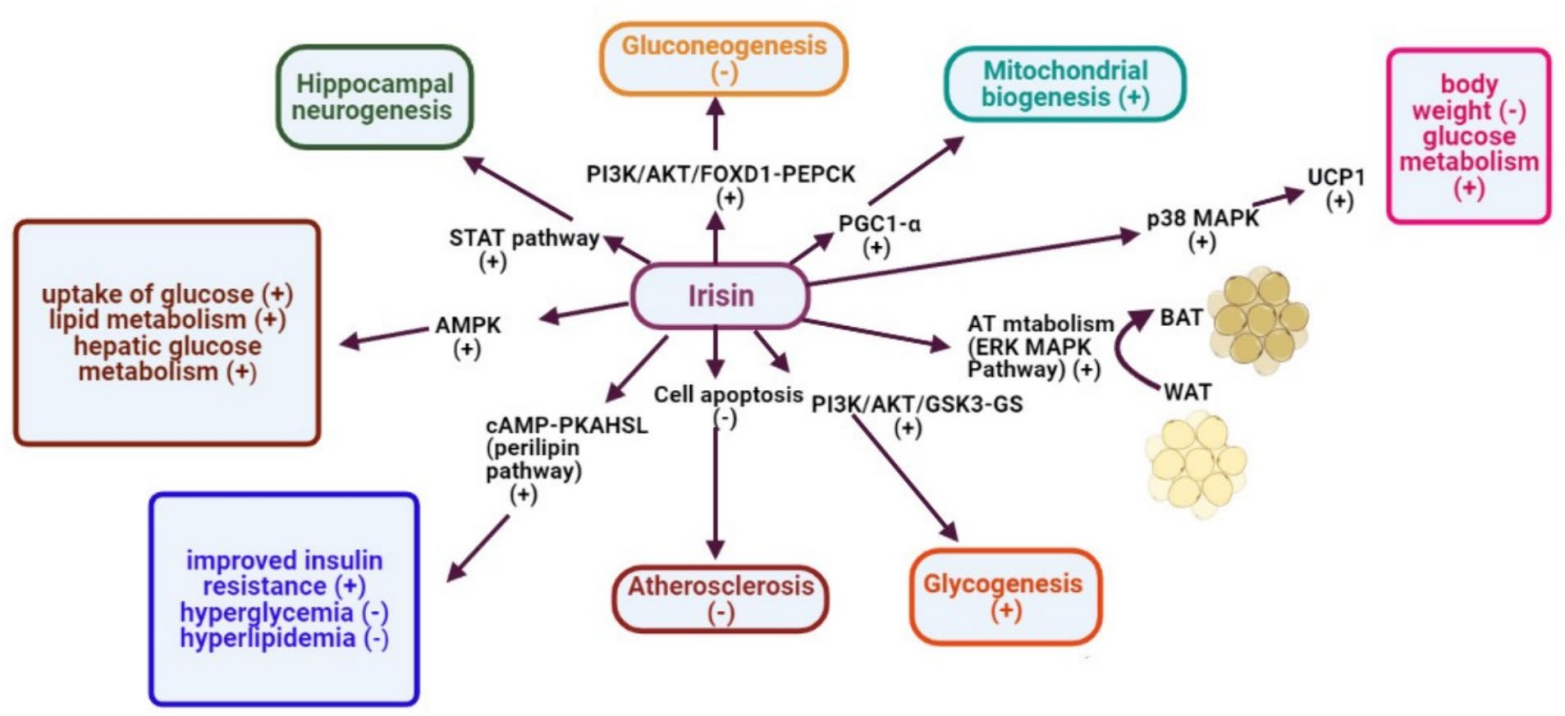
Activation of signaling pathways triggered by IRSN expression.

IRSN is a prominent adipocytokine deeply involved in various metabolic pathways, influencing lipid homeostasis, cardiovascular health, CNS processes, and overall energy metabolism (123, 124). It plays a central role in converting WAT to BAT, thus amplifying energy expenditure (125). A detailed depiction of the signaling pathways activated by IRSN is provided in Figure 4.

IRSN stimulates glycolysis and oxidative metabolism, endorsing mitochondrial biogenesis via key genes like Glut4 and UCP3, enhancing energy output, insulin sensitivity, and a healthier metabolic profile (126). These effects are mediated through various pathways, including cAMP-PKA-HSL (126).

### 10.2 Liver metabolism and thermogenesis

IRSN influences liver metabolism by stimulating key pathways and enzymes such as AMPK and LKB1, improving hepatic glucose and lipid metabolism (127, 128). The upregulation of UCP1 in BAT aids thermogenesis and energy expenditure, promoting weight loss and enhancing glucose metabolism (129).

### 10.3 Modulation by physiological changes

**Figure 5:**
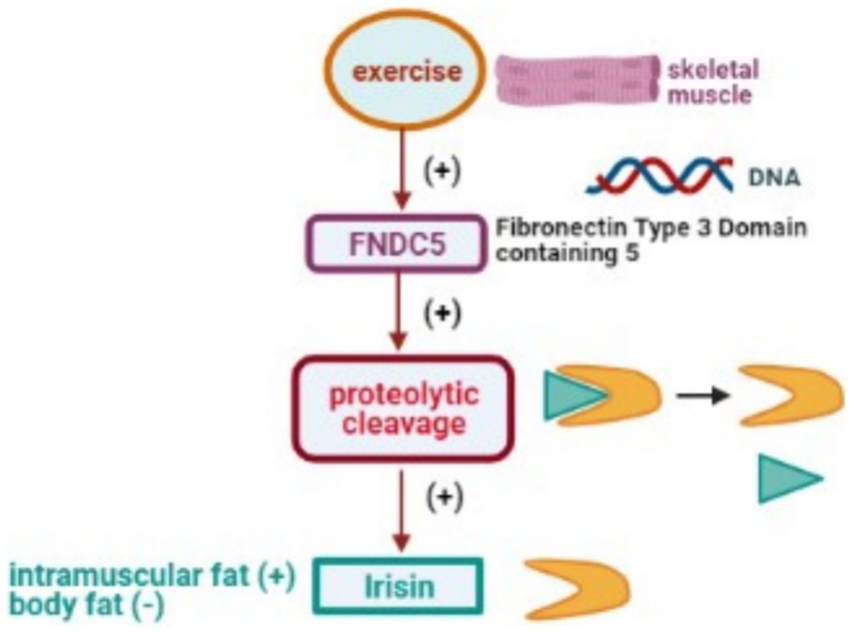
Physical activity stimulates DNA transcription of the FNDC5 gene, leading to proteolytic cleavage and subsequent release of the IRSN.

Physical activity significantly affects IRSN expression, with exercise increasing its plasma and skeletal muscle levels, and extended durations enriching its presence in the brain, potentially reducing anxiety (130). IRSN levels are also modulated by dietary adjustments, obesity, and specific pharmacological exposures (16). For a comprehensive overview of how physical activity impacts the transcription of the FNDC5 gene and leads to the release of IRSN, refer to Figure 5.

### 10.4 Implications for farm animals

In farm animals, IRSN shows intriguing responses. For example, camels exhibit a relationship between glucose and IRSN during exercise, suggesting regulatory roles of glucose, FFA, insulin, leptin, and cortisol on the PGC-1α/FNDC5/IRSN pathway (120). Postpartum dairy cows show correlations between FNDC5 plasma concentrations and various metabolic metrics, emphasizing IRSN’s importance during this period (15). In dairy cattle, IRSN plays a significant role in lipid metabolism, enhancing lipolysis and mobilizing lipid reserves post-parturition (15).

IRSN’s diverse roles in metabolic regulation and energy expenditure highlight its potential in improving health and productivity in farm animals. Understanding its pathways and effects can lead to innovative strategies for managing livestock metabolism.

Table 3 elucidates the biological effects of IRSN in laboratory animals and presents an all-encompassing summary of the observed results.

**Table 3:** Summary of findings: biological effects of IRSN in laboratory animal models.

Name	Dose	Species	Mode of administration	Major effect	Reference
Irisin	100 ng/kg/day	Obese rats	subcutaneously administered with osmotic minipumps	Irisin infusion rises asprosin levels in male rats (p¼0.02) but not in female rats. Irisin stopped the high glucose, low-density lipoprotein, triglyceride, and leptin levels caused by obesity in all groups, but it did not change the levels of asprosin in either obese male or obese female rats.	(131)
Recombinant irisin	2.8 μg and 4.3 μg	BALB/c mice	Intraperitoneal injections	When irisin is injected into the peritoneum of female BALB/c mice, the BMI, serum insulin, and LH levels drop significantly.	(132)
Irisin	1 μg/kg/day	male albino rats	Intraperitoneal injection	Irisin administered intraperitoneally dramatically improved platelet function, metabolic abnormalities induced by T2DM, and elevated sRAGE. Significantly negative associations were found between sRAGE and measures of platelet function and several glucometabolic variables.	(133)
Irisin	100 ng/mL/day	male Wister rats	intraperitoneal injection	The overall body weight of irisin and exercise-treated animals is considerably lower than that of the obese control group. Irisin injection or exercise reduces BMI, abdominal circumference, serum insulin, fasting glucose, Homa IR, osteopontin, TC, LDL, TG, FFA, and MDA by a significant amount.	(134)
Irisin	100 μg/kg/day	Male mice	intraperitoneal injection	Irisin injection increases brain metabolism by increasing ATP production.	(135)
Irisin	5 μL/rat	male SD rats	intraperitoneal microinjector	Irisin administered intraperitoneally through microinjection may modulate the expression of BDNF and glycometabolism in diabetic rats.	(136)
Irisin	0.55 nmol/μl, 1.45 nmol/day	Male C57/BL6J mice	Subcutaneous injection	Irisin subcutaneous injection improves glucose/lipid metabolic derangements and insulin resistance in obese mice while increasing lipolysis via the cAMP-PKA-HSL/perilipin pathway.	(126)

BMI, Body mass index; LH, Luteinizing hormone; TC, Total Cholesterol; LDL, Low-density lipoprotein; HDL, High-density lipoprotein; TG, Triglycerides; FFA, Free Fatty Acids; MDA, Malondialdehyde; ATP, Adenosine Triphosphate; BDNF, Brain-derived neurotrophic factor.

## 11 Roles of APLN, VSFTN, and IRSN in metabolic adaptations in farm animals

Farm animals, notably dairy cattle, undergo significant metabolic adjustments during early lactation. These shifts correlate with milk production and an observed disparity in feed intake, frequently leading to an NEB — a situation where energy demands outstrip consumption, with critical repercussions for post-calving health (137, 138). As they transition from late pregnancy to early lactation, there’s a marked decrease in insulin plasma levels, coupled with diminished insulin sensitivity in key tissues like skeletal muscle and AT (139). These alterations ensure glucose is adequately available for milk synthesis and fetal development, while heightened insulin resistance augments lipid mobilization, leading to elevated plasma NEFA and beta-hydroxybutyrate levels post-calving (140–144).

Adipokines, namely APLN, VSFTN, and IRSN, play instrumental roles in these metabolic transitions. Specifically, in dairy cows, APLN-36, a variant of APLN, exhibits elevated concentration and mRNA expression nearing parturition, showing an inverse relationship with serum NEFA levels. This suggests APLN’s potential to modulate lipid mobilization in dairy cows (51, 145, 146). VSFTN, with its critical function in insulin sensitivity and glucose regulation, gains prominence during the pregnancy-to-lactation transition, especially in high-producing dairy cows. Given its presence in mammary gland epithelial cells and milk, VSFTN might offer health benefits to calves (80, 147, 148). Conversely, IRSN is vital in lipid mobilization during post-partum NEB, with elevated plasma levels observed after calving (15).

In a study, diet-restricted calves displayed heightened VSFTN mRNA expression in liver biopsies versus controls, pointing to potential glucose metabolism irregularities and indicating the profound effect of diet on VSFTN expression and glucose regulation (97).

Furthermore, optimizing fat deposition in farm animals directly impacts meat quality. While some fats might be superfluous, intramuscular fat enhances flavor. The production of IRSN during physical activity could influence IM fat deposition, suggesting its potential to elevate meat quality (149).

The interplay between APLN, VSFTN, and IRSN in energy balance and metabolism, particularly in insulin resistance and obesity, is noteworthy. APLN levels are higher in obesity but drop with weight loss, while IRSN decreases in obesity but increases during exercise (150). Investigations have delved into the dynamics between these adipokines and metabolic parameters, revealing their fluctuating levels under normal, impaired glucose tolerance, and diabetic conditions (151). Unraveling these relationships and understanding their nuances is essential for the energy metabolism of farm animals and demands more in-depth exploration. The effects of the three adipokines on farm animals are summarized in Table 4.

**Table 4:** Summary of biological effects of APLN, VSFTN, and IRSN in farm animals.

Name	Species	Mode of administration	Major effect	Reference
APLN-13	Ewe	Intravenous bolus injections	Injection of APLN-13 increased the circulation level of several vasoactive hormones, including plasma arginine vasopressin, adrenocorticotrophin, aldosterone, cortisol, atrial and brain natriuretic peptide, cyclic GMP, and cyclic AMP with no effect on renal indices.	(60)
APLN-36	Dairy cows	Serum analysis	Decline in serum APLN-36 levels post-calving, closely associated with blood biochemical parameters indicative of the transition to a negative energy balance state.	(51)
APLN-13	Sheep	Serum analysis	APLN levels in ewes varied significantly based on breed, gender, and interactions with body condition score, but not by lactation or pregnancy status.	(42)
APLN-13	Sheep	Not specified	APLN’s ability to modulate molecules evident in its regulation of mammary gland activity during different physiological stages such as lactation and pregnancy.	(44)
APLN	Dairy cows	Milk analysis	APLN in cow’s milk offers nutritional and physiological benefits, emphasizing the milk’s value as a dietary component and potential modulator of energy regulation.	(51)
Recombinant VSFTN	Porcine adipocyte cells	Intraperitoneal immunization	VSFTN enhanced lipoprotein lipase expression in preadipocytes, facilitating lipid uptake and increased fatty acid synthase gene expression in differentiated adipocytes, which could enhance lipogenic activity. Moreover, VSFTN can upregulate IL-6 expression in pig adipocytes that have undergone differentiation.	(94)
VSFTN	Diet-restricted calves	Liver biopsy analysis	Heightened VSFTN mRNA expression in liver biopsies versus controls, indicating potential glucose metabolism irregularities and profound effects of diet on VSFTN expression and glucose regulation.	(97)
VSFTN	Pigs	Tissue analysis	Inverse correlation between VSFTN expression and body fat levels, with leaner pigs exhibiting higher VSFTN concentrations, raising questions about its reliability as an indicator of fat storage.	(98)
IRSN	Dairy cows	Serum analysis	Correlations between FNDC5 plasma concentrations and various metabolic metrics postpartum, emphasizing IRSN’s importance during this period.	(15)
IRSN	Holstein calves	Serum analysis	Delivery mode affects IRSN concentrations; dystocia and cesarean-born calves presented reduced IRSN levels post-colostrum intake.	(111)
IRSN	Dairy cattle	Serum analysis	IRSN concentrations heightened in cattle with subclinical ketosis compared to healthy counterparts; positive correlation with ghrelin levels.	(112)
IRSN	Dromedary camels	Exercise intervention	Relationship between glucose and IRSN during exercise, suggesting regulatory roles of glucose, FFA, insulin, leptin, and cortisol.	(120)
IRSN	Swine	Tissue analysis	IRSN localized in swine ovaries, suggesting implications for ovarian function.	(121)
IRSN	Arabian horses	Serum analysis	Serum IRSN levels linked to exercise regimes, indicating its potential role in energy regulation during physical activity.	(122)

Overall, APLN, VSFTN, and IRSN have pivotal roles in energy regulation in farm animals, especially during crucial metabolic transitions. Enhancing our understanding of these adipokines in farm animal contexts is fundamental for refining health and production strategies.

## 12 Energy regulation and reproduction in farm animals: effects of APLN, VSFTN, and IRSN

Farm animal fertility is intricately linked to energy metabolism. With the hypothalamus, anterior pituitary, and gonads collectively governing the reproductive system, energy status emerges as a determinant of fertility. In recent years, AT has evolved from being perceived merely as an energy storage site to an active endocrine organ, releasing a slew of adipokines. Notably, leptin and adiponectin have been pinpointed for their involvement in the hypothalamic–pituitary-gonadal axis and female reproductive tract. Yet, the adipokines APLN, VSTFN, and IRSN have garnered attention for their potential roles within various female reproductive systems of farm animals (20).

APLN and its receptor, APJ, are discernible in the ovaries across several farm animals, ranging from cattle to sheep (43, 152–155). Besides the ovaries, APLN is also detectable in sheep mammary glands (44). Furthermore, APLN has been identified in the uterus and uterine tubes as well as in the ovary (42). This adipokine has been found to augment steroidogenesis in several species, including cattle and porcine, and stimulate the proliferation of granulosa cells (152, 153, 156).

VSFTN’s presence is affirmed in the ovaries of animals like cattle, buffalo, chicken, and turkeys (89, 157–159). Its functional role varies across species; while it boosts steroidogenesis and granulosa cell proliferation in cattle, buffalo, and turkeys, its effect seems inverse in chickens (89).

IRSN’s landscape in farm animals is less explored. Though observed in porcine (121), its gene expression in cattle diverges from patterns seen in humans and mice (118). Preliminary data suggest the absence of IRSN expression in buffalo ovaries. IRSN is noted to influence steroidogenesis in porcine ovaries, but its broader implications for the reproductive systems of farm animals remain enigmatic.

Despite the evidence indicating adipokine involvement in certain species, data gaps exist, especially concerning their interactions with the hypothalamus-pituitary axis in farm animals. To realize the full scope of APLN, VSFTN, and IRSN’s roles in energy regulation and reproduction, targeted research in the context of farm animals is paramount.

## 13 Applications of APLN, VSFTN, and IRSN in farm animal production

In ruminants, such as sheep and goats, the role of APLN extends beyond mere energy regulation. It is intricately involved in postprandial responses and profoundly affects the secretion of growth hormone, arginine-vasopressin, and adrenocorticotropic hormones (160). Specifically, in sheep, APLN’s ability to modulate molecules is evident in its regulation of mammary gland activity. This regulation is characterized by the induction of hormonal activation and a biphasic hemodynamic response (60). During different physiological stages in ewes, such as lactation and pregnancy, serum APLN levels appear to be resilient to alterations in the body condition score (BCS). This consistent presence underlines its potential importance in energy regulation during these critical phases (161). Post-parturition, decreased levels of APLN are observed, suggesting its potential adaptive role during this period (146).

Dairy cows present another dimension to the understanding of APLN. Norvezh et al. (51) highlighted a decline in serum APLN-36 levels post-calving. This fluctuation is closely associated with blood biochemical parameters, indicative of the cow’s transition to an NEB state. Such alterations in APLN levels may be instrumental in helping dairy cows accommodate the increased energy requirements characteristic of lactation. Furthermore, cow’s milk represents a key dietary component for calves and humans. Rich in various adipokines, it offers both nutritional and physiological benefits. APLN, as a part of this adipokine profile, could be crucial for growth and overall health, emphasizing the milk’s value not just as a dietary component but as a potential modulator of energy regulation (162). Other findings in sheep showed that APLN levels in ewes varied significantly based on breed, gender, and their interactions with body condition score, but not by lactation or pregnancy status (161). These authors claimed that the variability in APLN levels during critical physiological phases and its potential role as a serum biomarker strongly suggest its potential application in diagnosing and understanding metabolic disorders (161). Similarly, APLN levels in dairy cows’ serum stayed stable between pregnancy and lactation, indicating slight fluctuation (52).

With the pivotal roles that APLN, VSFTN, and IRSN play in energy regulation across farm animals, it’s evident that understanding their dynamics can offer valuable insights. These insights can potentially drive advancements in farm animal production strategies, with health, growth, and reproductive efficiency implications.

## 14 Future research focus

Currently, there is a significant gap in knowledge regarding the specific roles of novel adipokines—APLN, VSFTN, and IRSN—in farm animals, particularly in terms of energy metabolism and production. Limited information is available on their impact, especially concerning VSFTN. Future research should aim to investigate the associations between these adipokines and their combined effects on energy regulation. Understanding these relationships will provide valuable insights into their physiological roles and potential applications for enhancing farm animal health and productivity.

Research priorities:

1. Interplay of adipokines: Investigate how changes in one adipokine influence the levels or functions of the others.
2. Impact on energy metabolism: Study the specific roles of APLN, VSFTN, and IRSN in energy metabolism in various farm animals.
3. Production outcomes: Assess the effects of these adipokines on farm animal production metrics, such as growth, milk yield, and meat quality.
4. Mechanistic studies: Conduct detailed mechanistic studies to understand the molecular pathways mediated by these adipokines.
5. Comparative analysis: Compare the roles and effects of these adipokines in farm animals with those observed in model animals to identify species-specific differences and similarities.

## Author contributions

BS: Conceptualization, Data curation, Formal analysis, Funding acquisition, Investigation, Methodology, Project administration, Resources, Software, Supervision, Validation, Visualization, Writing – original draft, Writing – review & editing. S-SJ: Writing - Review & editing HL: Writing – review & editing. HA: Writing – review & editing. AS: Writing – review & editing. AG: Writing – review & editing. SM: Writing – review & editing. SA: Writing – review & editing.
